# Faecal zonulin, calprotectin and the infant microbiome in early life

**DOI:** 10.1002/ctm2.1695

**Published:** 2024-05-21

**Authors:** Mickayla Bacorn, Poorani Subramanian, Shira Levy, Qing Chen, George L. Maxwell, Suchitra K Hourigan

**Affiliations:** ^1^ Clinical Microbiome Unit, National Institute of Allergy and Infectious Diseases (NIAID), National Institutes of Health (NIH) Bethesda Maryland USA; ^2^ Bioinformatics and Computational Biosciences Branch, Office of Cyber Infrastructure and Computational Biology, NIAID, NIH Bethesda Maryland USA; ^3^ Inova Women's Hospital Fairfax Virginia USA

Dear Editor,

Here, we present novel data associating faecal calprotectin and zonulin, biomarkers of intestinal inflammation and permeability respectively, and the gut microbiome during early life.

Early life is a critical window for the gut microbiome development, which educates immune and inflammatory regulation.^1^ Factors that occur during this window, including vaginal delivery and lack of antibiotic exposure, are known to be protective for health outcomes later in life.^1^

There is limited data regarding the infant microbiome as it relates to faecal zonulin and calprotectin. Zonulin is an endogenous protein that reversibly regulates tight junctions and indicates intestinal permeability.^2^ Calprotectin is present in neutrophils and other immune cells and indicates inflammation.^3^ These are well‐studied in the adult population and elevated levels are generally associated with disease.^2^, ^3^ Research shows that healthy infants have elevated zonulin and calprotectin without detectable disease.^4^, ^5^ We hypothesised that these biomarkers are positively associated with protective clinical factors and microbiome taxa during early life. Moreover, their presence may indicate immune maturation in infants as opposed to pathology. Therefore, we investigated the relationship between zonulin, calprotectin and the microbiome, as well as clinical factors known to influence the microbiome development and later health outcomes.

Participants of this study were enrolled in “The First 1000 Days of Life and Beyond” cohort. Two hundred twenty‐two stool samples from 53 infants, who were generally healthy without significant comorbidities, were collected at birth (meconium), 2 months (m), 6, 12, and 24 m with detailed demographic and clinical information. Biomarkers were quantified via ELISAs. Samples underwent DNA extraction and shotgun metagenomic sequencing performed on the Illumina HiSeq platform. Read pairs were trimmed for quality, classified taxonomically using Kraken, assembled using metaSPAdes, and annotated for functional pathways using HuMaNn3. Statistical comparisons were done at each timepoint separately with one sample per subject using MaAsLin2 for differential abundance of taxa and pathways, and Kruskal–Wallis tests for α‐diversity. See Supporting information for full method details.

We found that zonulin peaked at 6 m, while calprotectin was elevated at birth, with both subsequently decreasing over time (Figure 1A,B). At 2 m, higher zonulin levels were associated with vaginally delivered infants (*p* = .036), while at 6 m they were associated with no maternal antibiotic exposure (prenatal and/or peripartum) (*p* = .007) (Figure 1C). Increased calprotectin levels were associated at 2 and 6 m with no maternal antibiotic exposure (*p* = .042 and.034, respectively) (Figure 1D). Additionally, calprotectin was elevated in females at birth (*p* = .0002).

**FIGURE 1:**
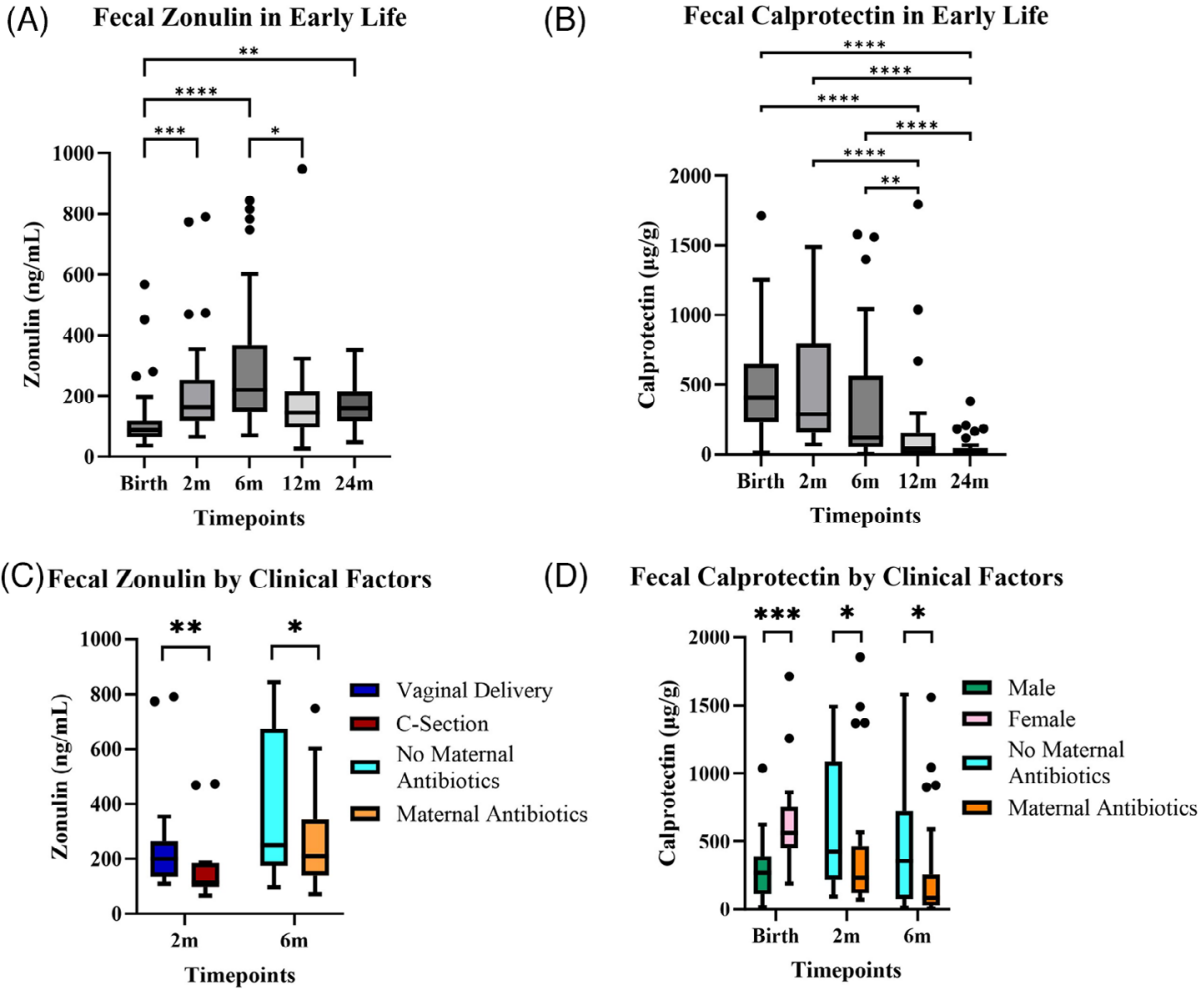
Zonulin and calprotectin are elevated during early life. Tukey box‐and‐whisker plots of (A) Zonulin over the first 2 years of life. (B) Calprotectin over the first 2 years of life. (C) Zonulin at 2 and 6 m stratified by the delivery mode and maternal antibiotic exposure. (D) Calprotectin at birth stratified by sex, and calprotectin at 2 and 6 m stratified by maternal antibiotic exposure. *p < .05; **p < .01; ***p < .001; ****p < .0001.

Shannon α‐diversity was positively associated with zonulin at 2 m (*p* = .002); there was no association between α‐diversity and calprotectin. β‐diversity did not differ with any biomarker quartiles.

Several bacterial taxa at the genus level and above were associated with these biomarkers (Figure 2A). At the species‐level, *Clostridioides difficile* positively associated with zonulin at 6 m (*p* = .001). Zonulin also positively associated with *Klebsiella quasipneumoniae* (*p* = .001) and negatively with *Rothia mucilaginosa* (*p* = .003) at 2 m, both of which stratified by delivery mode and/or no maternal antibiotic exposure (Figure 2B). Additionally, at the species‐level, calprotectin positively associated with several species of *Enterocloster* and *Clostridium* (*p* < .005) stratifying by the delivery mode and/or maternal antibiotic exposure at 6 m (Figure 2C). These associations were not seen at later timepoints. Regarding metabolic potential, zonulin positively associated with the following pathways at 6 m (*p* < .0005) stratifying by the delivery mode and maternal antibiotic exposure: pyruvate fermentation to butanoate (CENTFERM‐PWY), L‐glutamine biosynthesis III (PWY‐6549), and the superpathway of *Clostridium acetobutylicum* acidogenic fermentation (PWY‐6590) (Figure 2D).

**FIGURE 2:**
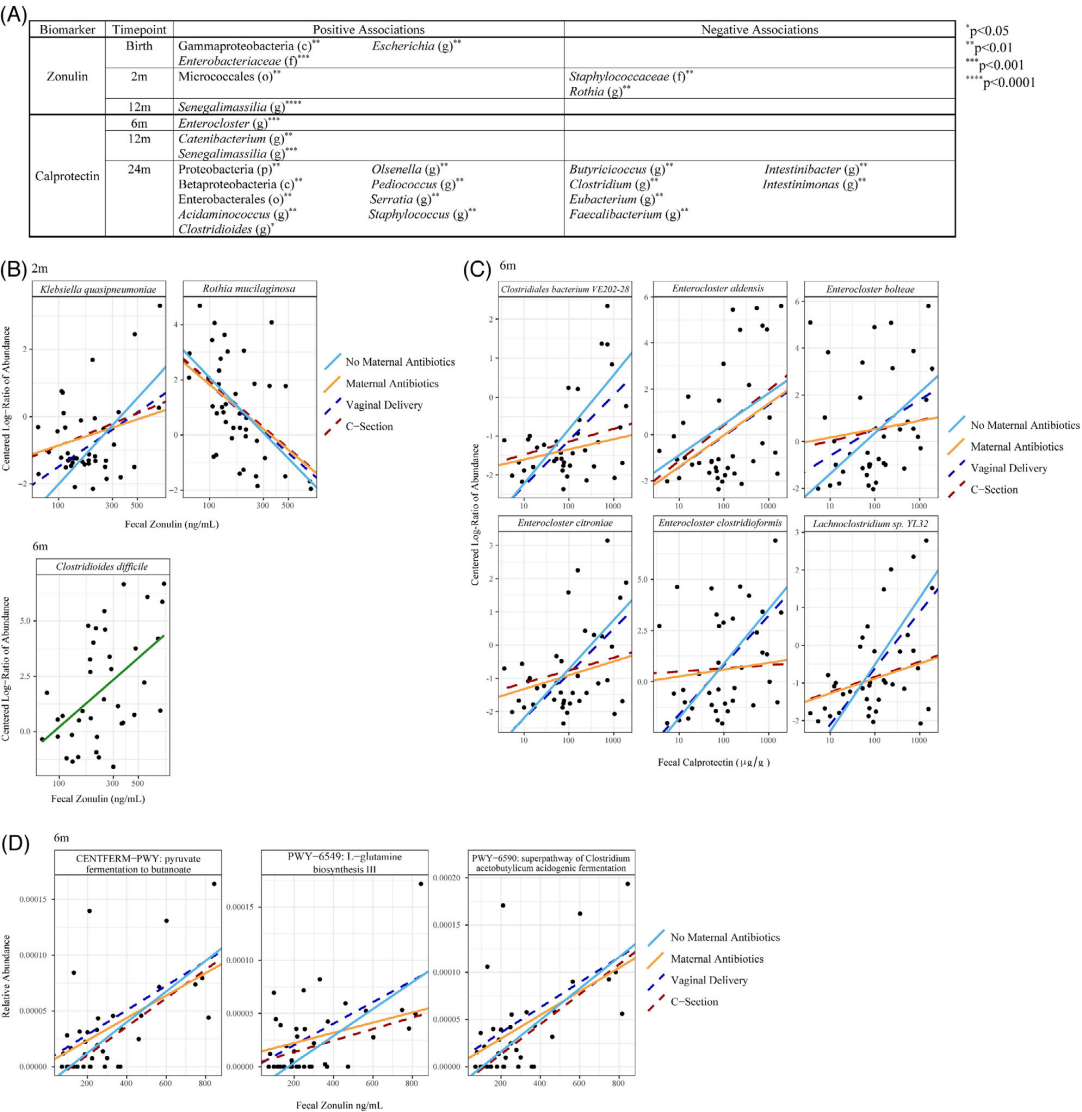
Bacterial taxa associations with zonulin and calprotectin levels at different timepoints. (A) Table of bacterial taxa at genus‐level or higher that associated with zonulin and calprotectin with a false discovery rate (FDR *q*‐value) less than.01 at various timepoints. The unadjusted *p*‐value is reported as asterisks. Taxa level is indicated as follows: p: phylum; c: class; o: order; f: family; and g: genus. (B) Graphs of centred log‐ratio of abundance of taxa at a species level found to significantly associate with fecal zonulin levels. (C) Graphs of centred log‐ratio of abundance of taxa at a species level found to significantly associate with fecal calprotectin levels. (D) Graphs of relative abundance for pathways found to significantly associate with fecal zonulin levels.

Building on the previous work, we observed that vaginal delivery and lack of maternal antibiotic exposure, which are known to positively influence the microbial and immune environment, were associated with higher zonulin and/or calprotectin levels at early timepoints only.^4^, ^5^ This suggests that these biomarkers play a different role during early life and may be related to healthy gut and immune development. It is worth noting that delivery mode and maternal antibiotic exposure are not mutually exclusive, as all caesarean sections in this study included the peripartum antibiotic treatment. Further, there are reports regarding low specificity of commercial ELISA kits for detecting faecal zonulin.

Furthermore, previous work uncovered that calprotectin was higher in vaginally delivered infants during the first week of life.^5^ We found a similar trend after birth but without statistical significance. However, zonulin was higher in vaginally delivered infants at the 2 m timepoint, supporting the idea that these biomarkers may indicate healthy gut development during early life. Interestingly, elevated calprotectin in females was also observed in a study of pre‐term infants during the first week of life.^6^ Our results suggest that the sex‐difference in calprotectin levels may not be restricted to pre‐term infants and resolve by 2 m of age.

Our novel use of shotgun metagenomics identified species‐level and metabolic pathway associations at early timepoints with zonulin and/or calprotectin which is previously unreported. Notably, *Clostridioides difficile* was positively associated with zonulin at 6 m. Although toxigenic *C. difficile* has pathogenic potential later in life, it is a normal component of the microbiome in infancy.^7^ The association with zonulin early in life could be postulated to support the immune maturation process. Moreover, *Rothia mucilaginosa* was negatively associated with zonulin at 2 m. *R. mucilaginosa* is associated with the oral microbiome and can produce microbiome altering enterobactin.^8^ Thus, *R. mucilaginosa* may alter the gut environment triggering reduced zonulin levels. Additionally, zonulin positively correlated at 6 m with short‐chain fatty acid (SCFA) fermentation pathways and stratified by delivery mode and maternal antibiotic exposure. SCFA fermentation is an indicator of gut health and shapes the gut mucosal immune system.^9^, ^10^ We hypothesise that the positive correlation with zonulin may contribute to immune education at this early life timepoint.

In conclusion, elevated zonulin and calprotectin were associated with various bacterial taxa, as well as early life clinical factors known to have an important influence on health outcomes later in life. Taken together, we hypothesise that these faecal biomarkers may be related to healthy microbiome and immune development during this critical period as opposed to being associated with pathology as in adults,^2^, ^3^ with exposure to certain microbes and their inflammatory mediators influencing immune system education. Further investigation to examine this is warranted given this preliminary observation.

## AUTHOR CONTRIBUTIONS

*Concept and design*: Mickayla Bacorn, Shira Levy, Suchitra K Hourigan. *Acquisition, analysis, or interpretation of data*: All authors. *Drafting of the manuscript*: Mickayla Bacorn *Critical revision of the manuscript for important intellectual content*: All authors. *Statistical analysis*: Mickayla Bacorn, Poorani Subramanian. *Obtained funding*: George L. Maxwell, Suchitra K Hourigan. *Supervision*: Suchitra K Hourigan. All authors reviewed and approved the manuscript.

## CONFLICT OF INTEREST STATEMENT

The authors declare no conflicts of interest.

## FUNDING INFORMATION

National Institute of Allergy and Infectious Diseases of the National Institutes of Health under the Intramural Research Program (SKH). The content is solely the responsibility of the authors and does not necessarily represent the official views of the National Institutes of Health. Inova expresses its appreciation to the Fairfax County in VA, which has supported Inova's research projects with annual funding from its Contributory Fund (Fund 10030, Maxwell). This work also used the Office of Cyber Infrastructure and Computational Biology High Performance Computing cluster at NIAID, Bethesda, MD.

## ETHICS APPROVAL

This study was Institutional Review Board approved (Inova protocol #15‐1804, WCG protocol #20120204) with written informed consent obtained.

## Supporting information

Supporting information

## Data Availability

The datasets generated and/or analysed in this study are available in the NCBI SRA repository under BioProject number: PRJNA988496. https://www.ncbi.nlm.nih.gov/bioproject/PRJNA988496
