# Supplementary material for: Faecal zonulin, calprotectin and the infant microbiome in early life
Source: Clin Transl Med. 2024 May 21;14(5):e1695. doi: 10.1002/ctm2.1695 (PMC11109039; doi:10.1002/ctm2.1695)
Supplement: Supplementary file 1 — Supporting information [file CTM2-14-e1695-s001.docx]

**SUPPORTING INFORMATION**

**Methods Overview**

**
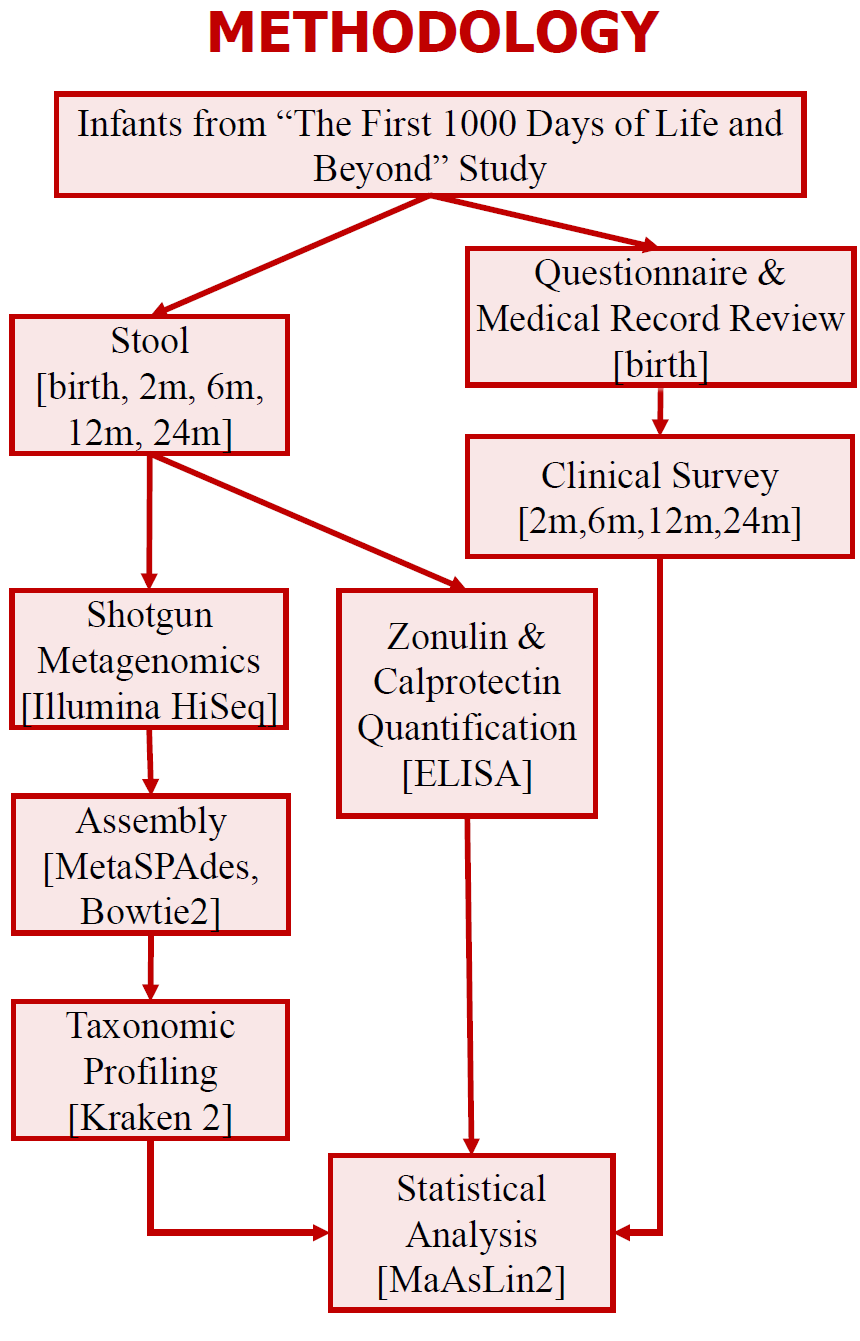
**

**Study Design**

Samples were collected from an Institutional Review Board approved longitudinal, prospective cohort study “The First 1000 Days of Life and Beyond” (Inova protocol #15-1804, WCG protocol #20120204) within the Inova Health System in Northern Virginia, USA. Participants of this study were enrolled prenatally with informed consent, and then provided serial stool samples from their infants at birth (meconium) and at around 2 months(m), 6m, 12m, and 24m of age as described in previous publications^1, 2^. The meconium sample was collected at the hospital and the remaining samples were collected at home on sterile cotton swabs and mailed to the lab following a previously validated method for storage at -80°C until use^3^. At each timepoint, detailed demographic and clinical information were collected including delivery mode and antibiotic use. Subjects for this study were selected based on availability of stool at all time points.

**Stool Sequencing**

*Shotgun Metagenomics*

DNA was extracted from stool via the DNeasy PowerSoil Pro kit (Qiagen, Valencia, CA) following the kit’s instructions. Shotgun metagenomic sequencing was performed on the Novaseq platform (Illumina, CA, USA). Positive controls (DNA sequences) and negative controls (DNA free water) were used.

*Assembly*

Read pairs were trimmed for quality at Q20 with BBDuk v38.0.1^4^ and then assembled using metaSPAdes v3.14^5^ with default parameters. Reads were mapped back to the assembly using Bowtie2 v2.4.4.

*Taxonomic Profiling and Functional Annotation*

Taxonomic profiling of the raw reads was done with Kraken v2.1.2^6^ and a custom database made from the GTDB release 202^7^ collection of bacterial genomes, viruses and eukaryotes from the standard Kraken database with additional genomes from the EuPathDB Kraken index. Gene finding of the metagenomes was done using Prodigal v2.6.3^8^ on the assemblies, and gene read counts were computed with VERSE v0.1.5^9^. Functional annotation of the genes and pathway abundance estimation was done with HuMaNn v3.0.0.

**ELISA**

*Fecal Calprotectin*

Fecal calprotectin was assessed using the Buhlmann Fecal Calprotectin ELISA kit (BÜHLMANN fCAL® ELISA, <https://buhlmannlabs.com/buhlmann-fcal-elisa/>). The kit protocol for extraction was adapted to accommodate stool swabs. Stool weight was quantified using a standardized swab weight. Extraction process was increased to one hour and validated using lab standards.

*Fecal Zonulin*

Fecal zonulin was assessed using the Immundiagnostik Fecal Zonulin ELISA kit (IDK® Zonulin (Stool) ELISA, <https://www.immundiagnostik.com/en/testkits/k-5600>). Previously extracted stool proteins were applied to the kit protocol.

**Statistical Analysis**

*Clinical Factors and Biomarkers*

Biomarker data at each timepoint was evaluated for normal distribution via Shapiro-Wilk tests. All failed (p<0.05) except zonulin at 24m. Timepoints were then stratified by clinical factors of interest and evaluated for statistical differences via Mann-Whitney tests with a p-value cutoff of 0.05 for significance.

*Metagenomics*

Differential abundance of taxa and pathways was assessed using MaAsLin2 v1.15.1 R package^10^. Taxonomic abundances (read counts) were first transformed by centered-log ratio and then fitted with the linear model *~ Zonulin*. For associations with delivery mode and maternal antibiotics usage, the models *~ Zonulin * Delivery Mode* and *~ Zonulin * Maternal Antibiotics* respectively were used. Pathway abundances were normalized using HuMaNn’s^11^ copies per million before being fit with the same models. The analogous statistics were done for the comparisons associated with calprotectin. Beta diversity was analyzed using the Bray-Curtis measure. Samples were separated into groups according to quartiles of the associated values of calprotectin and zonulin using the quantile function in R. These groups were compared using a PERMANOVA test using the adonis2^12^ function from the vegan R package v2.6^13^ with the formulae *~ Zonulin_quartile*, *~ Zonulin_quartile * Delivery Mode* , and *~ Zonulin_quartile * Maternal Antibiotics*

**References**

1. Mani, J.*, et al*. Epidemiological and microbiome associations of Clostridioides difficile carriage in infancy and early childhood. *Gut Microbes*. **15**, 2203969 (2023).

2. Nashed, L.*, et al*. Gut microbiota changes are detected in asymptomatic very young children with SARS-CoV-2 infection. *Gut*. **71**, 2371-2373 (2022).

3. McDonald, D.*, et al*. American Gut: an Open Platform for Citizen Science Microbiome Research. *mSystems*. **3**, 10.1128/msystems.00031-00018 (2018).

4. Bushnell, B. BBMap. 2023.

5. Nurk, S., Meleshko, D., Korobeynikov, A. & Pevzner, P.A. metaSPAdes: a new versatile metagenomic assembler. *Genome Res*. **27**, 824-834 (2017).

6. Wood, D.E. & Salzberg, S.L. Kraken: ultrafast metagenomic sequence classification using exact alignments. *Genome Biology*. **15**, R46 (2014).

7. Parks, D.H.*, et al*. GTDB: an ongoing census of bacterial and archaeal diversity through a phylogenetically consistent, rank normalized and complete genome-based taxonomy. *Nucleic Acids Research*. **50**, D785-D794 (2021).

8. Hyatt, D.*, et al*. Prodigal: prokaryotic gene recognition and translation initiation site identification. *BMC Bioinformatics*. **11**, 119 (2010).

9. Zhu, Q., Fisher, S.A., Shallcross, J. & Kim, J. VERSE: a versatile and efficient RNA-Seq read counting tool. *bioRxiv*. 053306 (2016).

10. Mallick, H.*, et al*. Multivariable association discovery in population-scale meta-omics studies. *PLOS Computational Biology*. **17**, e1009442 (2021).

11. Franzosa, E.A.*, et al*. Species-level functional profiling of metagenomes and metatranscriptomes. *Nature Methods*. **15**, 962-968 (2018).

12. Anderson, M.J. A new method for non-parametric multivariate analysis of variance. *Austral Ecology*. **26**, 32-46 (2001).

13. Oksanen, J.*, et al*. vegan: Community Ecology Package. R package version 2.6-4. 2022.
